# Surface Characteristics of Thermally Modified Bamboo Fibers and Its Utilization Potential for Bamboo Plastic Composites

**DOI:** 10.3390/ma15134481

**Published:** 2022-06-25

**Authors:** Fu Hu, Lifen Li, Zhigang Wu, Liping Yu, Baoyu Liu, Yan Cao, Hailong Xu

**Affiliations:** 1 College of Forestry, Guizhou University, Guiyang 550025, China; Hufu2655@163.com (F.H.); wzhigang9@163.com (Z.W.); ylpgzu@163.com (L.Y.); 2 Special and Key Laboratory for Development and Utilization of Guizhou Superior Bio-Based Materials, Guizhou Minzu University, Guiyang 550025, China; liubaoyu1020@163.com (B.L.); 02190707@163.com (Y.C.); xu00hailong@163.com (H.X.)

**Keywords:** vacuum heat treatment, bamboo fiber, polyethylene, bamboo plastic composite, physical and mechanical properties

## Abstract

Bamboo fibers are considered as a more attractive option for the reinforcement of wood plastic composites as compared to wood fiber due to its fast growth rate and good toughness. Heat treatment is an environment-friendly method of improving the integrated performance of bamboo materials. This paper highlights the heat treatment of bamboo fiber for suitable properties as reinforcements in bamboo plastic composites. The effects of vacuum heat treatment on the surface characteristics of bamboo fibers and the properties of bamboo plastic composites were analyzed by studying the chemical composition, surface elements and polarity of bamboo fiber before and after treatment, and the physical and mechanical properties of bamboo plastic composite. The results showed that after vacuum heat treatment, the bamboo fibers became darker and experienced a transition from green to red. Moreover, FTIR, XPS and contact angle analysis indicated that the hemicellulose content, the oxygen/carbon ratio and the polar component of the bamboo fiber had a decreasing trend as the treatment temperature increased. In addition, the 24 h water absorption and the 24 h thickness expansion rate of the water absorption showed a trend of first decreasing and then increasing as the treatment temperature increased, while the bending performance of bamboo plastic composite showed a trend of increasing first and then decreasing as a result of increased treatment temperature. Therefore, a combined process of vacuum heat treatment and the addition of MAPE could improve the physical and mechanical properties of bamboo plastic composites to a certain extent.

## 1. Introduction

Over the past few decades, attempts have been made to replace synthetic fiber with nature fibers in fiber reinforced composites, due to the distinct and specific properties of nature fibers such as low cost, lightweight, renewability, and biodegradable. The application of natural fibers can cover various types of filling matrices, such as ceramics, cement, concrete [1] and polymers [2], behaving as a reinforcement material.

Among natural fibers, bamboo has received extensive attention as a promising reinforcement for matrices because of its excellent mechanical properties [3]. Furthermore, bamboo is widely regarded as sequestering carbon due to their fast growth rate [4]. However, the utilization rate of bamboo in the traditional bamboo industry is not high, only about 40% [5]. In order to realize the resource utilization of bamboo waste, bamboo plastic composite materials prepared by hot pressing, extrusion injection molding and other ways with plastics are widely used in construction, packaging, automotive interior and other fields due to their excellent performance, such as dimensional stability, water resistance, wear resistance, corrosion resistance and ease of secondary processability. However, the poor compatibility between hydrophilic bamboo fibers and hydrophobic thermoplastic matrix seriously affects the physical and mechanical properties of bamboo plastic composites [6,7]. In order to improve the interfacial compatibility between the bamboo fiber and the plastic matrix, many recent researchers spend a lot of effort in the surface treatment of bamboo fiber by various physical and chemical means [8,9], such as alkali treatment, esterification modification and graft modification treatment of plant fibers or addition of compatibilizers. However, these methods still have problems, such as complicated process, high cost and poor practicability [10,11,12].

High temperature heat treatment is a simple and environmentally friendly method that could improve the interface compatibility of wood plastic composites without the addition of any chemical reagents. During the treatment process, the wood fibers are treated in an anaerobic or low-oxygen environment such as steam, hot oil or inert gas, and processed at high temperatures for a period [13]. Among them, heat treatment with vacuum as a medium is relatively simple, and it causes little change in wood color compared with other medium, which provides feasibility for the application of plant fiber in composite. During heat treatment, hemicellulose with poor thermal stability and high water absorption in plant fibers would be degraded, which in turn reduces the hygroscopicity of plant fibers and improves the dimensional stability of wood fiber-thermoplastic resin composite [14,15,16,17]. In addition, high temperature heat treatment is also a green environmental protection method that improves the corrosion resistance of wood, due to the degradation of chemical components such as hemicellulose, which reduce the number of hydrophilic hydroxyl group [18,19,20].

In recent years, there have been many studies on the high temperature modification of wood fiber in wood plastic composites by referring to the high temperature heat treatment method of wood, and those studies have mainly focused on the influence of heat treatment on dimensional stability, mechanical properties, decay resistance and weathering properties of wood plastic composites [21,22]. The surface characteristics of wood fiber have an important influence on the physical and mechanical properties of wood plastic composites [23], but at present, the structure-function relationship between the surface properties of the heat-treated fibers and the physical and mechanical properties of heat-treated wood plastic composites is rarely reported. Therefore, in this study, vacuum heat treatment was used to modify bamboo fibers. The vacuum heat-treated bamboo fibers/polyethylene (HTBF/PE) composites were prepared by hot pressing, and the effect of the heat treatment on the water resistance and mechanical properties of wood plastic composites was analyzed from the perspective of fiber surface properties, including functional groups and surface polarity, which provided a theoretical basis for the preparation of high-performance HTBF/PE. 

## 2. Materials and Methods

### 2.1. Materials

Moso bamboo processing residues were purchased from Chishui, Guizhou Province, and 40–60 mesh bamboo fibers were screened and oven-dried at 80 °C until a constant weight in an air-blast drying oven before vacuum treatment. Polyethylene powder (PE-600, 100–150 mesh) was obtained from Suyuan Plastic Material Mall, Zhangtoumu Town, Dongguan, Guangdong Province. Maleic anhydride grafted polyethylene (MAPE) was purchased from Shanghai Macklin Biochemical Co., Ltd. (Shanghai, China), with a grafting rate of 8%.

### 2.2. Preparation of the Composites

(1) Modification of Bamboo Fiber by Vacuum Heat Treatment

The oven-dried bamboo fibers were placed into the vacuum drying box, the vacuum pump was turned off when the vacuum reached −0.08 MPa, and the temperature was raised to 100 °C for 30 min, and then warned up to 140, 160, 180, 200 and 220 °C, respectively. Timing began when the temperature reached the specified temperature, and it was held for 2 h. During the heat treatment, the vacuum degree was maintained at −0.08 MPa. After the heat treatment, the heating device of the vacuum heat treatment was turned off, the air was deflated, and then the specimen was taken out when the oven temperature dropped to about 60 °C.

(2) Preparation of Bamboo Plastic Composites

The vacuum heat-treated bamboo fibers, PE and MAPE, were evenly mixed according to the mass ratio of 40:56:4, and then paved in a self-made mold. The composite was prepared by hot pressing in a flat vulcanizing machine at a temperature of 180 °C for 8 min in the pressure range from 5 to 7 MPa, and the design density of bamboo plastic composite was 1.1 g/cm^3^.

### 2.3. Performance Testing and Characterization

#### 2.3.1. Surface Characteristics Analysis of Modified Bamboo Fibers

(1) Color measurement: The bamboo flour before and after the heat treatment was pressed into pellets by a tableting machine, and the color parameters L*, a*, b* were recorded with CM-2003d colorimeter according to the CIELAB color system. Five points were taken for each sample, and the results were taken as the average value. In addition, the ΔL*, Δa*, Δb* were calculated using the unheated samples as a reference, and the corresponding total color change (ΔE*) was calculated using the following Equation (1).
  (1) $$\Delta E*=\sqrt{\Delta L{*}^{2}+\Delta a{*}^{2}+\Delta b{*}^{2}}$$
(2) Fourier Transform Infrared Spectroscopy (FTIR): The FTIR spectra of the bamboo fiber before and after heat-treated were determined in the range of 4000–400 cm^−1^ by an average of 32 scans at a resolution of 4 cm^−1^.
(3) X-ray Photoelectron Spectroscopy (XPS): The XPS spectra were obtained using the Thermo Scientific K-Alpha X-ray photoelectron spectrometer (Thermo Fisher Scientific, Waltham, MA, USA) equipped with a Al K_α_ X-ray source. The survey and high-resolution scans were performed at 100 eV pass energy with 1 eV step and 50 eV pass energy with 0.05 eV step, correspondingly.
(4) Contact Angle Test: The contact angle of the bamboo fiber before and after the heat treatment was determined using a contact angle measuring instrument with distilled water and ethylene glycol as the test liquid. Owens–Wendt method was used to calculate the surface free energy through Equations (2) and (3). The polar component and dispersion component of distilled water is 51 mJ/m^2^ and 21.8 mJ/m^2^, respectively, while the polar component and dispersion component of ethylene glycol is 19 mJ/m^2^ and 29 mJ/m^2^, respectively.
  (2) $$\gamma_{1}\left(1+\cos\theta \right)=2\left(\sqrt{\gamma_{s}^{p}\gamma_{1}^{p}}+\sqrt{\gamma_{s}^{h}\gamma_{1}^{h}}\right)$$
  (3) $$\gamma_{w}=\gamma_{s}^{p}+\gamma_{s}^{h}$$
  where, $\gamma_{1}$ is the surface energy of the test liquid, *θ* is the contact angle between the test liquid and the fiber, $\gamma_{1}^{p}$ is the polar component of the surface energy of the testing liquid,$\;\gamma_{1}^{h}$ is the dispersion component of the surface energy of the test liquid, $\gamma_{s}^{p}$ is the polar component of the surface energy of the fiber, $\gamma_{s}^{h}$ is the dispersion component of the fiber surface energy, and $\gamma_{w}$ is the surface free energy of fiber.

#### 2.3.2. Performance Test of Bamboo Plastic Composite

(1) Water Resistance Tests: The contact angle of the composites at 1 s was measured using a JY_PH300ML contact angle measuring instrument (Shanghai Zhongchen Digital Technology Equipment Co., Ltd., Shanghai, China), and the water absorption and thickness swelling of bamboo plastic composite for 24 h was tested according to the national standard GB/T 17657-2013.
(2) Mechanical Properties Tests: The bending performance of bamboo plastic composite was conducted according to GB/T 9341-2008, the testing speed was set up at a rate of 1.9 mm/min, and the span was 64 mm. The impact performance was tested according to GB/T 1043 -2008, the testing speed was set up at a rate of 3.8 m/s, and the impact energy of the pendulum was 7.5 J.
(3) Thermogravimetric (TG) Analysis: The temperature range of thermal cracking was 100–800 °C under nitrogen atmosphere at the heating rate of 10 °C/min.

**Figure 1:**
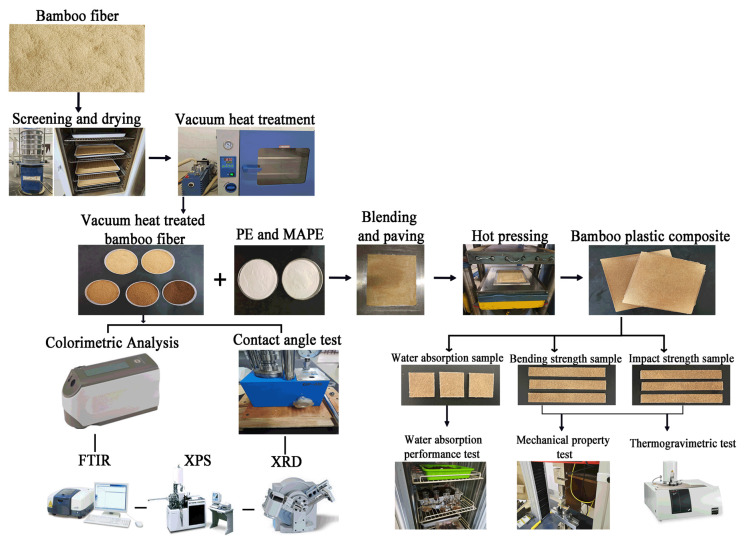
The preparation flow chart of vacuum heat-treated bamboo fiber/polyethylene composites.

In summary, the preparation and testing process of the vacuum heat-treated bamboo fiber/polyethylene composites is described in Figure 1.

## 3. Results and Discussion

### 3.1. Effect of Vacuum Heat Treatment on Surface Properties of Bamboo Fibers

#### 3.1.1. Effect of Vacuum Heat Treatment on Surface Color of Bamboo Fibers

**Figure 2:**
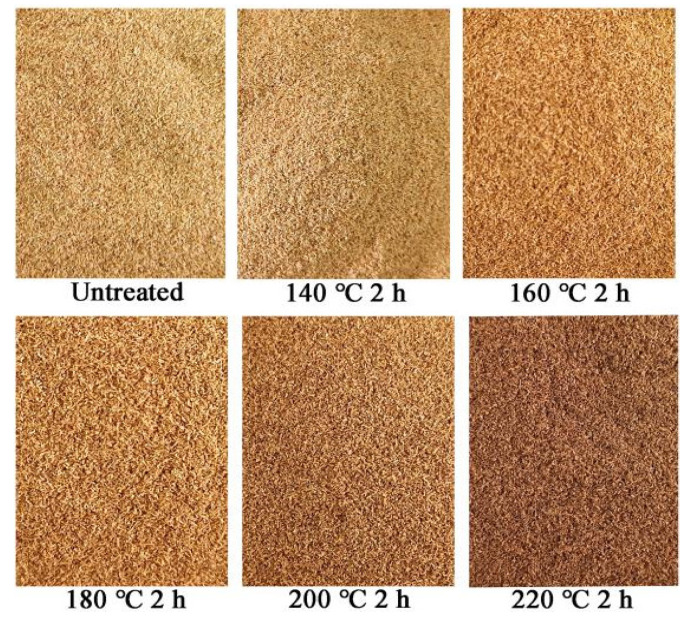
Color change of bamboo fiber after vacuum heat treatment.

Figure 2 shows the appearance of bamboo fibers before and after heat treatment at different temperatures for 2 h, and the color parameters of wood fiber pellets can be seen in Table 1. Here, L* represents the lightness from 0% (black) to 100% (white), a* represents the red hue from green (−*a*) to red (+*a*), and b* represents the yellow hue from blue (−*b*) to yellow (+*b*). According to Figure 2 and Table 1, as the vacuum heat treatment temperature increased, the heat treatment induced a significant decrease in L* values from 74.56 to 49.34, an increase in redness (a* values) from 2.64 to 6.97, while b* values showed a tendency of first increasing and then decreasing, which indicates that after the vacuum heat treatment the bamboo fibers became darker and experienced the transition from green to red. Moreover, the total color change (parameter Δ*E*) ranged from 2.14 to 25.65, implying that the color change of the heat treatment bamboo fibers above 160 °C could be clearly seen by the human eye as an obviously different color (Δ*E* *> 5*) [24]. The increase of chromophore groups and the decrease of auxochromic groups in the bamboo fibers are the fundamental reasons for the deepening of their color [25]. The specific reasons are mainly that, on the one hand, polysaccharides such as the cellulose amorphous area and the unstable hemicellulose in the bamboo fibers were degraded by heat under high temperatures. Indeed, furfural and other phenolic compounds with chromophore groups are more easily generated with the increase of heating temperature [26]. However, with the increase of temperature, the relative content of lignin increased. According to the literature reports [26], the lignin content in bamboo was increased from 21.51% to 38.47% when treated at 210 °C for 2 h, which increased the conjugation system and extended the absorption spectrum to the visible region, resulting in a deepening of the color of the bamboo fibers [27,28].

#### 3.1.2. Effect of Vacuum Heat Treatment on the Surface Polarity of Bamboo Fiber

The degree of polarity difference between wood fibers and plastic matrix is directly related to the interfacial binding performance of wood plastic composite [29]. In order to analyze the surface polarity of the bamboo fibers before and after heat treatment, distilled water and ethylene glycol were used as probe liquids to test their contact angles on the surface of the bamboo fibers, the surface free energy, polar component and dispersion component of the bamboo fibers under different treatment conditions were calculated, and the results are shown in Table 2. As can be seen from Table 2, with the increase of the vacuum heat treatment temperature, the surface free energy of the bamboo fiber shows the tendency of first decreasing and then increasing, and the surface free energy of the bamboo fiber reached the minimum when the treatment temperature was 160 °C, which was 33.64 mJ·m^−2^. When the heat treatment temperature ranged from 140 °C to 180 °C, the surface free energy of the bamboo fibers is reduced, which is due to the degradation of the main chemical components of the bamboo fiber to varying degrees after proper heat treatment, and the number of hydroxyl groups with strong hydrophilicity on the surface of the fiber surface was reduced, resulting in a decrease in fiber surface polarity and surface free energy. This not only reduces the hygroscopicity of the bamboo fiber, but also helps to improve the interfacial bonding between the bamboo fibers and plastic matrixes [30]. When the heat treatment temperature was higher than 200 °C, the surface free energy of the bamboo fiber was increased, which agreed with the results of Gérardin et al. [31,32], and the dispersion component of the wood surface free energy was increased after the heat treatment at high temperature, while the polar component is strongly reduced, indicating that the vacuum heat treatment could improve the interfacial compatibility between the bamboo fiber and PE matrix, and the higher the heat treatment temperature, the better the interfacial affinity. 

#### 3.1.3. Effects of Vacuum Heat Treatment on the Functional Groups of Bamboo Fibers

**Figure 3:**
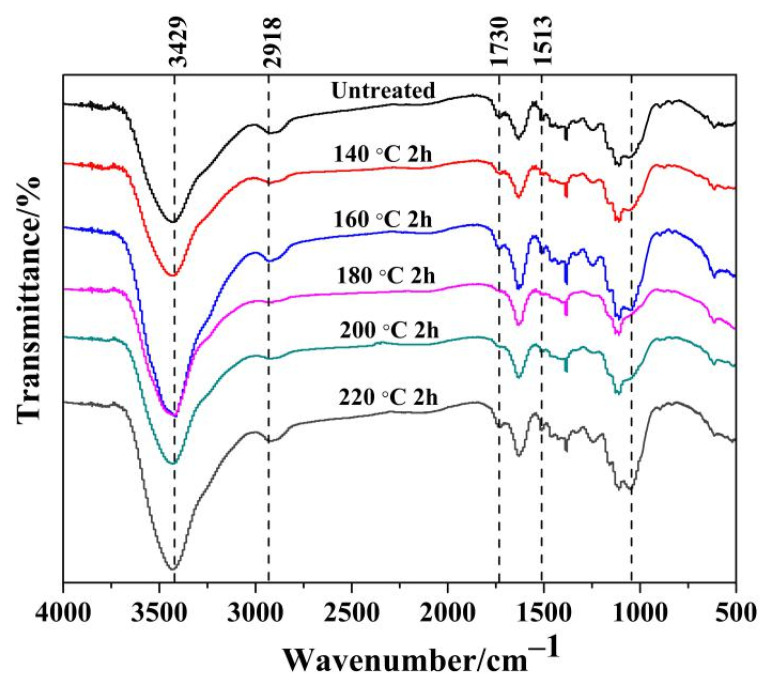
Infrared spectrum of bamboo fiber treated in vacuum.

FTIR was used to analyze the changes of bamboo chemical functional groups before and after heat treatment, and the results are shown in Figure 3. The absorption peaks at 3429 cm^−^^1^ and 2918 cm^−^^1^ correspond to the expansion and contraction vibration of the hydroxyl group the and C-H stretching symmetric vibration peaks of the methyl group, respectively, and both the absorption peak of the bamboo fibers before and after the heat treatment did not change significantly. The characteristic band at 1730 cm^−^^1^ is assigned to the stretching vibration of the carbonyl group (C=O) acetyls in the hemicellulose. As the heat treatment temperature increased from 140 to 180 °C, the band intensity showed a decreasing trend, indicating that the heat treatment caused the cleavage of acetyl groups in the hemicellulose. Additionally, a slight increase in the intensity of the carbonyl band was observed when the temperature reached 200 °C, which may be caused by the formation of new carbonyl groups by esterification reactions. These results were consistent with the previous reported findings by Gao et al. [33] and Yang et al. [34]. Furthermore, the bands at 1 632 cm^−1^ and 1514 cm^−^^1^ are the stretching vibration of the conjugated carbonyl group (C=O) and the vibration peak of aromatic ring skeleton of lignin, respectively [35].

#### 3.1.4. Effect of Vacuum Heat Treatment on the Surface Element Composition of Bamboo Fiber

**Figure 4:**
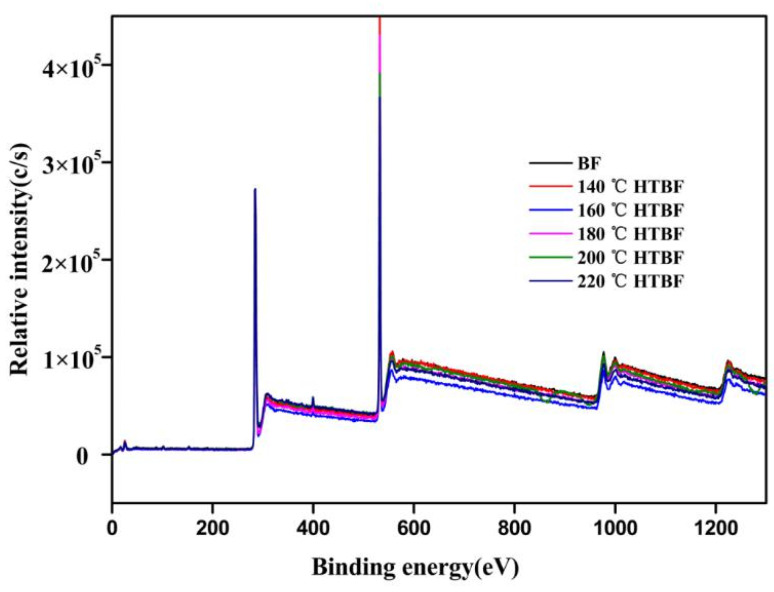
XPS wide-scan spectra of the heat-treated bamboo fiber surface.

In order to analyze the surface properties of the bamboo fibers before and after vacuum thermal treatment, the element composition of the bamboo fiber surface was analyzed by X-ray photoelectron spectroscopy (XPS), and the XPS wide scan spectra are shown in Figure 4. It can be seen that all XPS wide scan maps have strong peaks near 285 eV and 532 eV, indicating that the surface of the bamboo fiber contains a large number of C and O elements. In addition, there is a weak absorption peak near 400 eV, implying that the surface of the bamboo fiber also contains a small amount of N element. The element content and oxygen/carbon ratio on the surface of the bamboo fiber before and after the vacuum heat treatment are shown in Table 3. Note that with the increase of vacuum heat treatment temperature, the oxygen/carbon ratio on the surface of the bamboo fiber shows a gradual decreasing trend, which is consistent with the analysis results of the surface elements of heat-treated wood fiber [36]. The reduction of oxygen/carbon ratio is mainly due to the degradation of the hemicellulose and part of the cellulose during the heat treatment, which leads to the increase of lignin content. In addition, by-products with lower oxygen content were produced under heat treatment conditions, due to the dehydration of polymers in the wood or the formation of new components from the degraded substances, which also reduce the oxygen/carbon ratio.

**Figure 5:**
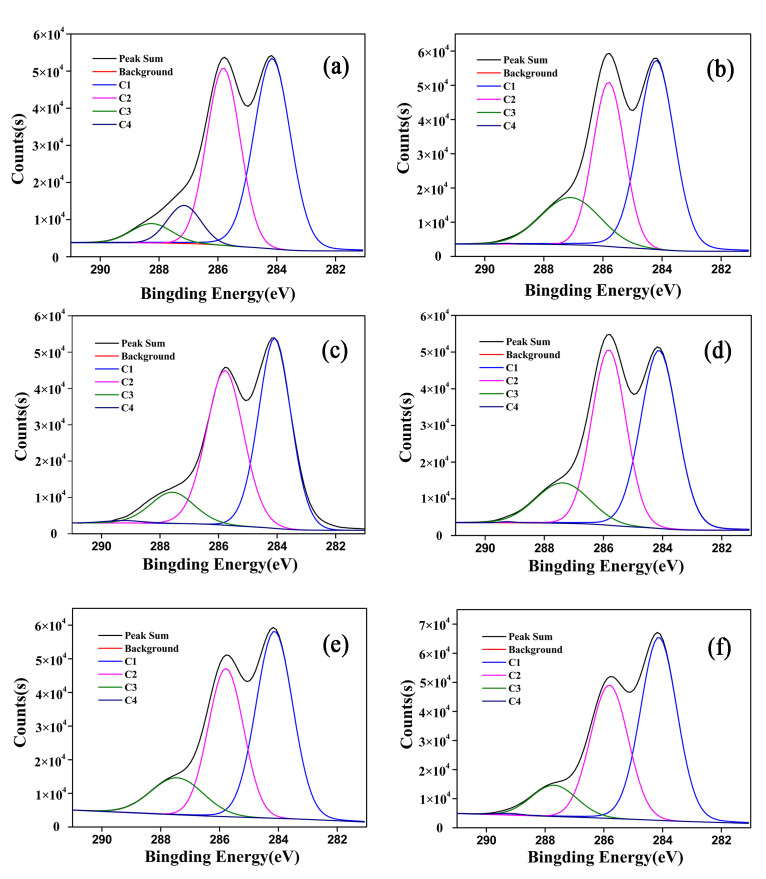
C1s XPS spectra of untreated and vacuum heat-treated bamboo fiber surface, wherein (**a**–**f**) are C1s XPS spectra of untreated, 140 °C, 160 °C, 180 °C, 200 °C and 220 °C, respectively.

To gain a deeper understanding of various functional groups on the surface of the bamboo fibers, the C1s signal could usually be divided into four components according to the number of oxygen atoms bonded to C, so as to obtain the chemical structure information of wood surface. The XPS peak software was used to curve-fit the C1s peak and divided it into four peaks, indicating that there were four forms of carbon on the bamboo surface. In this case, C1 (284.82 eV) corresponds to the carbon atoms bound only to carbon atoms or hydrogen atoms, and the corresponding functional groups are –C–C or –C–H, mainly including hydrocarbon compounds from the phenylpropane unit of lignin and fatty acids. C2 (286.46 eV) corresponds to the carbon atoms bound to an oxygen atom, and the functional group is –C–O single bond, mainly C–O–H in the cellulose and the hemicellulose molecule. C3 (288.07 eV) corresponds to a carbon atom bound to one carbonyl or two non-carbonyl oxygen atoms, and the functional group is –C=O, mainly derived from aldehydes, ketones and acetals in the bamboo. C4 (289.25 eV) is associated with the carbon atoms bonded to the carbonyl or non-carbonyl oxygen atoms, and the functional group is mainly O–C=O, mainly from the hemicellulose, carboxylic acids and ester groups in the extract. Generally, the C1 and C4 components are mainly from lignin and extracts, and the C2 and C3 components are mainly from carbohydrate [37]. The XPS C1s spectra of the bamboo fibers before and after modification were fitted into four peaks, and the results are shown in Figure 5, while the positions and relative content of C1, C2, C3 and C4 are shown in Table 4. 

As can be seen from Table 4, after the heat treatment, the relative content of C1 peaks showed a trend of first decreasing and then increasing, and the relative content of C1 on the surface of the untreated bamboo fiber and the heated-treated bamboo fibers decreased from 47.72% to 47.37%, 46.85% and 45.11%, and then increased to 49.62% and 51.58% at 140, 160, 180, 200 and 220 °C, respectively. The relative content of C1 reduced first, implying that the relative content of the lignin and resin extracts on the fiber surface decreased after the heat treatment. Subsequently, the relative content of C1 peak increased, possibly due to the thermal decomposition of hemicellulose, which is consistent with the chemical composition and functional group analysis of the fibers at 1514 cm^−1^. The relative content of C2 peak showed a trend of decrease in general, indicating that the relative content of the hemicellulose and part of the cellulose on the surface of the bamboo fiber reduced after heat treatment, which was due to the poor thermal stability of the hemicellulose and its susceptibility to degradation during heat treatment. The relative content of C3 peak increased overall, indicating that the content of carbonyl functional groups on the fiber surface increased after heat treatment, possibly due to the fact that part of the hydroxyl groups on the fiber surface were oxidized to carbonyl groups under heat treatment conditions, and lignin would undergo esterification reaction to generate carbonyl groups under heat treatment conditions [38], which is consistent with the results of FTIR analysis. In addition, the relative content of C4 showed an overall decreasing trend, which was caused by thermal degradation reactions in hemicellulose and part of the extracts during heat treatment.

### 3.2. Effect of Vacuum Heat Treatment on Physical and Mechanical Properties of Bamboo Fiber/Polyethylene Composites

#### 3.2.1. Effects on Physical Properties

1. Water resistance analysis

**Figure 6:**
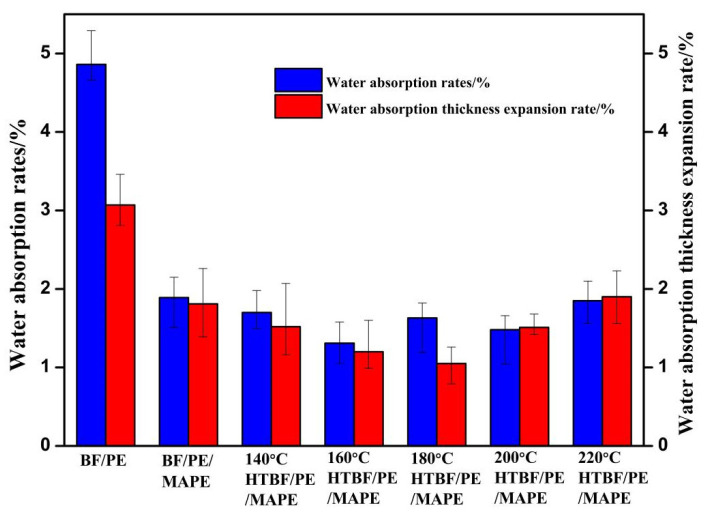
Influence of vacuum heat-treated bamboo fiber on the water absorption and the water absorption thickness expansion rate of bamboo plastic composite.

Figure 6 shows the influence of the vacuum heat treatment of the bamboo fiber on the water absorption and the water absorption thickness expansion rate of the composites. The addition of MAPE significantly reduces the water absorption and the water absorption thickness expansion rate of the composites. Compared with the control group (4.86% and 3.07%), the water absorption and the water absorption thickness expansion rate of the composite material decreased to 1.89% and 1.81%, respectively. The water resistance improved, mainly due to the reaction of the anhydride group on MAPE with the hydroxyl group on the bamboo fiber, forming an ester bond that improves the hydrophobicity of the bamboo fiber [39] and reduces the fiber polarity, thereby improving the interface bonding between the bamboo fiber and the PE matrix, and further improving the water resistance of the composite material [40].

In addition, the temperature of the vacuum heat treatment also affects the water resistance of the composites. When the treatment temperature was 160 °C, the water absorption reached the minimum value of 1.32%, and when the treatment temperature was 180 °C, the water absorption thickness expansion rate was the minimum, which was 1.05%. The results showed that the heat treatment could also reduce the water absorption and the water absorption thickness expansion rate of the composites, effectively improve its dimensional stability, and reduce the probability of deformation. This is mainly because the hemicellulose component, especially the polysaccharide glucosides, undergoes chemical changes after the heat treatment to form the monomers with weak hygroscopicity, and the hydroxy groups in the cellulose molecular chain combine with each other to form a hydrogen bond. In addition, in the process of heat treatment, the bamboo fiber microfilaments would move closer to each other, and when the distance between microfibrils became less than a certain distance, chemical bonds would form between the hydroxyl groups on adjacent microfibrils. In this way reducing the hydroxyl group content could reduce the hydroscopic capacity of the bamboo fibers [16]. However, when the heat treatment temperature was too high, cellulose and other substances were degraded to generate a large number of short-chain molecules, which reduce the strength of the bamboo fiber itself and weaken the water resistance of the composites. This is in keeping with reports in the previous literature that crystalline cellulose begins to degrade at 180 °C, while the hydrolysis reaction rate of cellulose increases significantly with reaction temperature [41].

Table 5 shows the contact angle and surface free energy of BF/PE composites. As can be seen with the increase of the vacuum heat treatment temperature, the contact angle of the composites first increases and then decreases, implying that the surface free energy shows a trend of first decreasing and then increasing. When the temperature was 180 °C, the surface free energy of the composite material reached the minimum of 41.73 mJ·m^−2^, indicating that the stability of the composite material was the best, which was consistent with the results of the water absorption thickness expansion rate of the composites.

2. Thermal stability analysis

**Figure 7:**
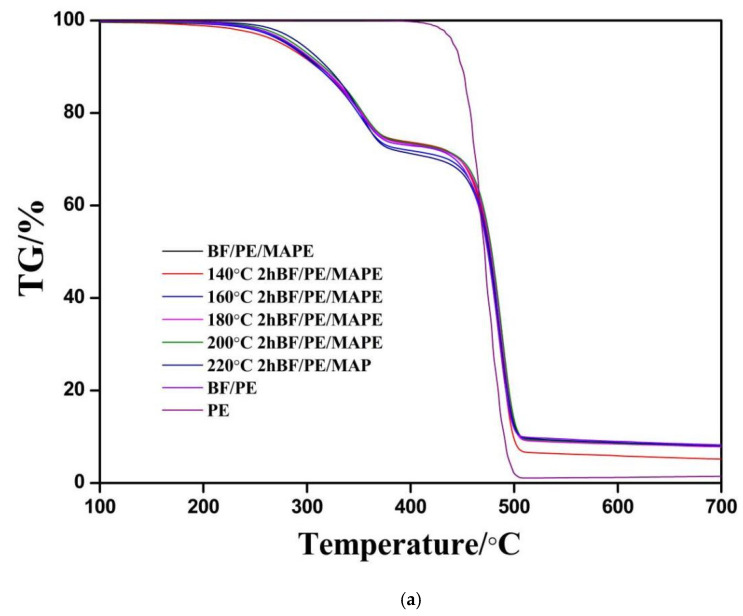

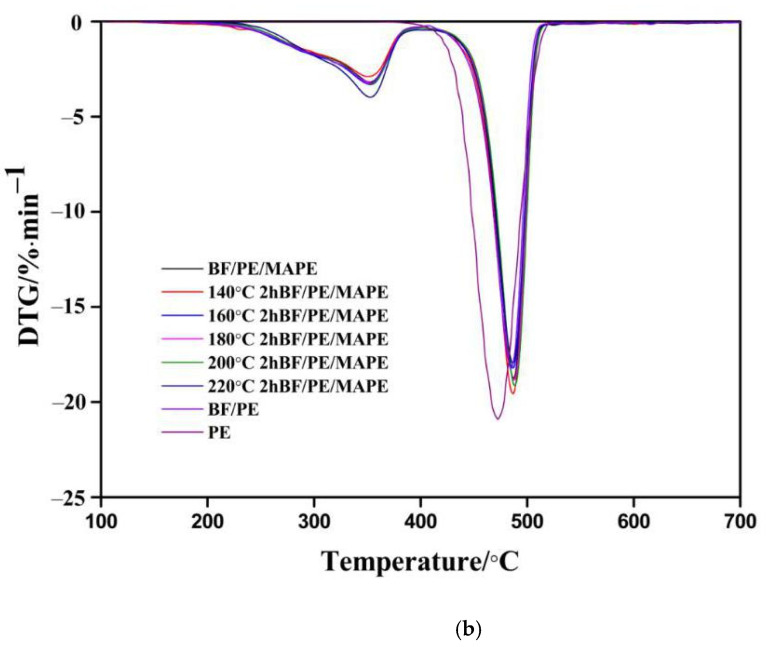
TG (**a**) and DTG (**b**) curves of bamboo plastic composite.

To analyze the effect of the heat treatment on the thermal stability of PE and bamboo plastic composite, the mass retention rate and mass loss rate of PE and the bamboo plastic composite under the conditions of 100~700 °C were analyzed by thermogravimetric weight, and the results are shown in Figure 7 and Table 6. As can be seen from Figure 7 and Table 6, the pyrolysis process of bamboo plastic composite could be roughly divided into three stages. The first stage was 100~200 °C, mainly caused by the evaporation of water and pyrolysis of a small amount of hemicellulose, and the weight loss is not obvious at this stage. The second stage was 200~400 °C, which was mainly caused by the weight loss of hemicellulose, cellulose, part of lignin in the bamboo fiber and polyethylene with relatively small molecular weight. The third stage was 400~700 °C, which was mainly caused by the pyrolysis of the residual lignin in the bamboo fibers and polyethylene with relatively high molecular weight [42,43]. Compared with the untreated bamboo fiber/PE composite, the vacuum heat treatment of the bamboo fiber could improve the thermal stability of the composite to a certain extent, for example, the onset thermal degradation temperature of the composite prepared from the bamboo fiber treated at 220 °C increased from 293.9 °C to 301.8 °C and 464.3 °C to 466.6 °C at stage 2 and stage 3, respectively, but the overall improvement effect is not obvious. In addition, the neat PE begins to decompose at 451.7 °C and ends at 489 °C with very little residue, and the amount of char residue at 700 °C was only 1.57%. In general, the addition of the bamboo fiber significantly reduces the thermal stability of PE at 200~400 °C but increased the char residual of PE at 700 °C to a certain extent.

#### 3.2.2. Effect of Heat Treatment on the Mechanical Properties of Composites

(1) Bending performance

**Figure 8:**
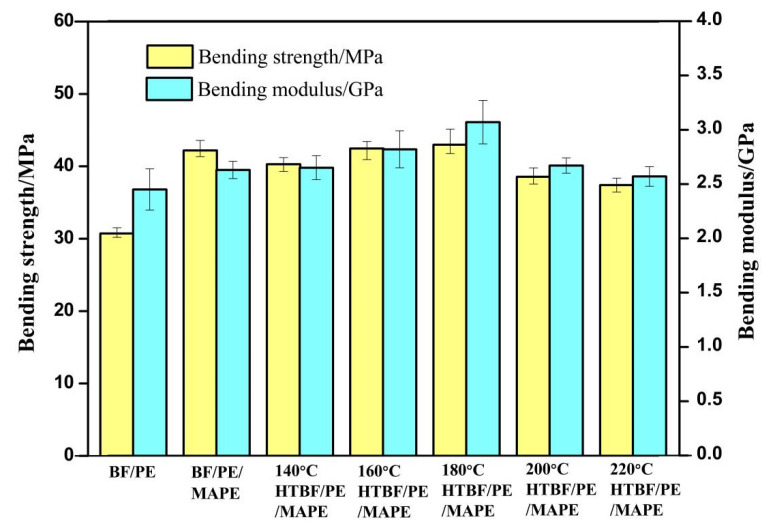
Bending strength and bending modulus of bamboo plastic composites.

**Figure 9:**
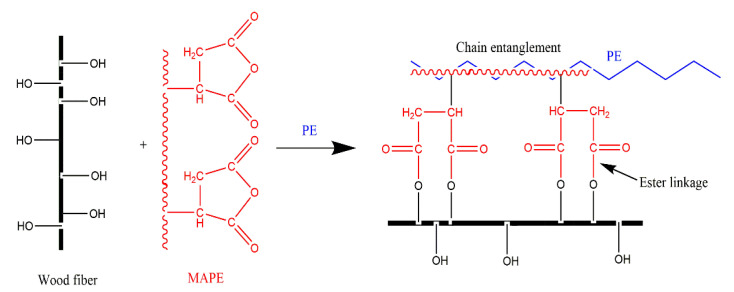
Schematic diagram of interaction in wood/PE/MAPE.

Figure 8 shows the bending strength and bending modulus of PE and HTBF/PE composites. The bending strength and bending modulus of BF/PE and BF/PE/MAPE were 30.72 MPa, 2.45 GPa, and 42.19 MPa, 2.65 GPa, respectively. Compared with BF/PE, the addition of MAPE increases the bending strength and bending modulus by 11.47 MPa and 0.20 GPa, respectively. The improvement of bending properties may be due to the formation of strong ester bonds between the maleic anhydride moiety of MAPE and the hydroxyl of wood fibers, which enhances the compatibility between the hydrophilic fibers and the hydrophobic PE matrix; moreover, the chain entanglement between PE and MAPE could also improve the bending properties of the composite (as shown in Figure 9) [44,45].

Heat treatment of the bamboo fibers could also improve the bending performance of composites to a certain extent. Figure 8 shows that when the heat treatment time is the same, with the increase of the heat treatment temperature, the bending strength and bending modulus of the composite material showed a trend of first increasing and then decreasing, and when the temperature was 180 °C, the bending strength and bending modulus of the composites reach the maximum, which was 42.98 MPa and 2.98 GPa, respectively. This is mainly due to three reasons. First, with the increase of the heat treatment temperature, the degradation of the hemicellulose components caused the cellulose microfibrils to aggregate with each other, and the microfibrils after polymerization would have a larger cross section size and a higher rigidity, which is the main reason for the improvement of some mechanical properties of the heat-treated bamboo fiber under mild treatment condition [46]. Second, according to the analysis of the influence of the heat treatment on the surface element composition of the bamboo fiber, when the heat treatment condition was 160 and 180 °C, the relative content of C1s peak component C2 was the largest, indicating that the relative content of the cellulose is the largest, thereby increasing the esterification between wood fibers and MAPE [23]. Third, with the increase of the heat treatment temperature, the polar component reflecting the polarity value of the fiber surface was gradually reduced, which means that the higher the heat treatment temperature, the better the interface affinity between the modified bamboo fiber and non-polar plastic.

When the bamboo fiber was treated at higher temperature (over 200 °C), the bending properties of the composite become worse. This may be due to two reasons: on the one hand, the hemicellulose would degrade to form formic acid and acetic acid after heat treatment, and these degradation products would further promote the degradation of cellulose amorphous region, lignin, and lignin-carbohydrates complex. This would result in the destruction of the fiber cell wall structure, and consequently the strength of the bamboo fiber would be reduced, ultimately leading to a decrease in the flexural strength of the composites [47]. On the other hand, XPS analysis showed that the content of the C1s peak component increased at 200 °C and 220 °C, implying that the relative content of lignin on the surface of the bamboo fiber increased; the increase of lignin concentration would affect the esterification reaction between fiber and MAPE and inhibit the formation of ester bond, thereby reducing the mechanical properties of composite [48]. The phenomenon that high temperature heat treatment may lead to the reduction of the bending strength of wood and wood composite materials has also been reported in previous studies [49].

(2) Impact performance

**Figure 10:**
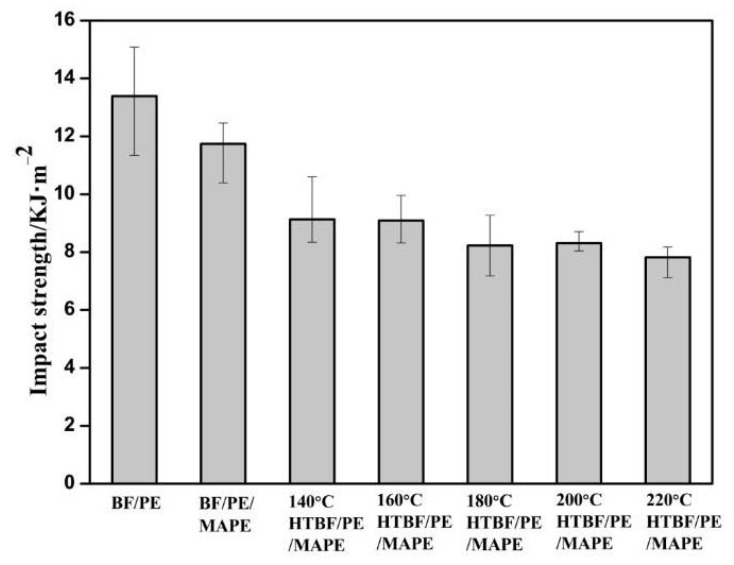
Impact strength of bamboo plastic composites.

As can be seen in Figure 10, the addition of MAPE reduced the impact strength of bamboo plastic composite to a certain extent. This may be due to the increased compatibility of the composite after the addition of MAPE, resulting in the increased brittleness of the composite, which leads to the fracture mode of the composite changed from “fiber pull-out” to “fiber breakage.” Compared to brittle fiber breakage, the former one requires a large amount of energy during the crack propagation [50].

With the increase of the heat treatment temperature, the impact strength of the composites shows a decreasing trend on the whole, which may be due to the degradation of the hemicellulose and part of the cellulose at higher temperatures. This led to the strength of the bamboo fiber itself and the number of hydroxyl groups decreasing to a certain extent, which in turn weakens the strength of the esterification reactions between the bamboo fiber or PE and MAPE. As a result, the impact performance of the composites was weakened [23]. In addition, new defects would be formed between the crystalline phase and the amorphous phase of the cellulose during the heat treatment process, which would also lead to the reduction of impact strength, and the decrease in impact strength was more pronounced at the higher heat treatment temperature [22].

## 4. Conclusions

This work intended to determine the potential of the heat-treated bamboo fiber for preparing bamboo plastic composite. The results showed that the vacuum heat treatment could reduce the hemicellulose content and the surface polarity of the bamboo fibers, thereby improving the interface compatibility between the bamboo fiber and PE matrix. When the heat treatment temperature was 160 °C, the 24 h water absorption of the composite was reduced by 73.01% compared with the control group, and when the temperature was 180 °C, the 24 h thickness expansion rate of the water absorption of the composites reached the minimum, which was 71.47% lower than that of the control group. Moreover, with the increase of the vacuum heat treatment temperature, the mechanical strength of the composites showed a trend of first increasing and then decreasing, and when the temperature was 180 °C, the bending strength and bending modulus of the composites both reached the maximum value, which were increased by 39.91% and 21.77%, respectively, as compared with the control group. Accordingly, the results indicated that combining the coupling agent and the heat treatment could significantly improve the water resistance and bending strength of the composites, while it may reduce the impact strength of bamboo plastic composite to a certain extent. 

Overall, the present work provides insights into the potential valorization of the heat-treated bamboo fiber for the reinforcement of polymer composite and shows that there is a certain relationship between the surface properties of the heat-treated bamboo fiber and the physical and mechanical properties of the heat-treated bamboo plastic composite. Further work should focus on the optimization of the heat treatment process of the bamboo fiber in order to prepare high-performance bamboo plastic composite. Additionally, research on the anti-corrosion, the insect-resistance and the durability of the heat-treated bamboo plastic composite must be carried out to improve its application in outdoor fields such as building materials, plank roads and pavilions, etc.

## Figures and Tables

**Table 1:** Color parameters of vacuum heat-treated bamboo fiber.

Sample Type	Surface Lightness (L*)	Red–Green Axis Chromatic Index (a*)	Yellow–Blue Axis Chromatic Index (b*)	Total Color Change (ΔE*)
Untreated	74.56 ± 0.79	2.64 ± 0.19	10.30 ± 0.75	—
140 °C 2 h	73.09 ± 0.80	3.05 ± 0.14	11.80 ± 0.29	2.14
160 °C 2 h	70.53 ± 0.92	3.71 ± 0.33	14.95 ± 1.13	6.24
180 °C 2 h	65.89 ± 0.86	5.24 ± 0.23	14.04 ± 1.25	9.79
200 °C 2 h	58.70 ± 0.83	6.41 ± 0.30	13.12 ± 0.57	16.54
220 °C 2 h	49.34 ± 1.09	6.97 ± 0.39	8.55 ± 0.43	25.65

**Table 2:** Surface contact angle and free energy of moso bamboo fiber.

Sample Type	Untreated	Vacuum Heat Treatment Temperature/°C				
		140	160	180	200	220
Water contact angle/°	53.00	62.50	72.50	83.00	85.50	86.00
	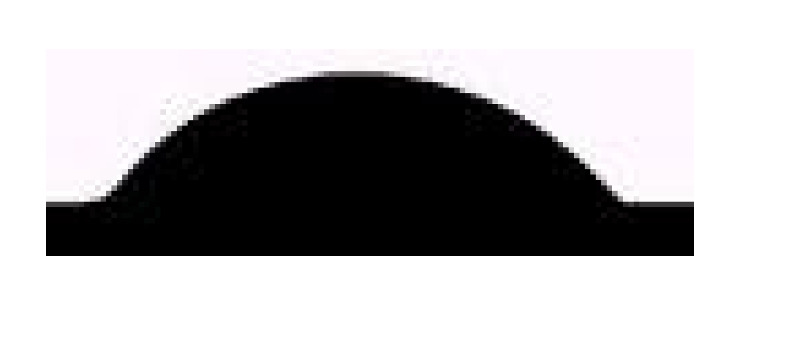	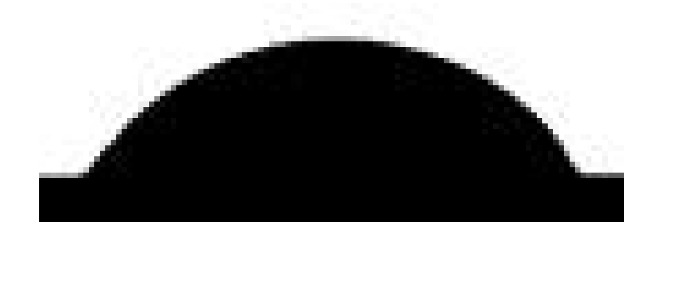	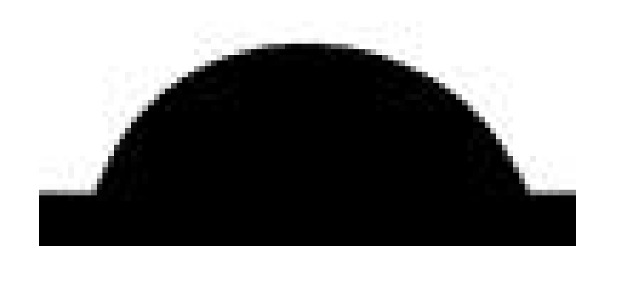	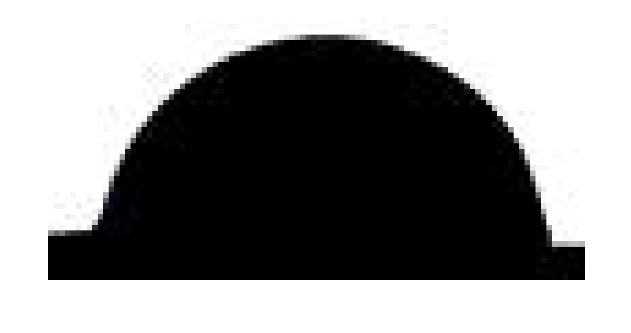	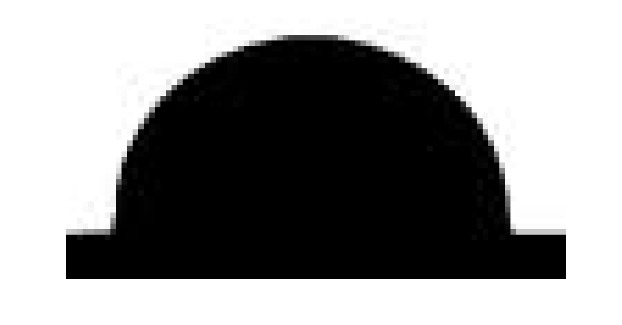	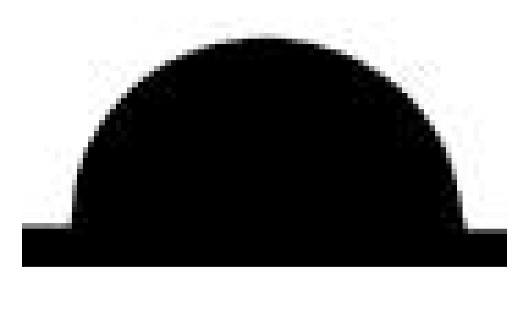
Ethylene glycol contact angle/°	37.00	42.00	47.00	48.00	42.00	41.50
	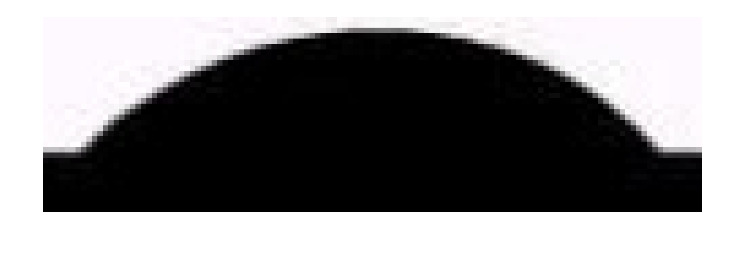	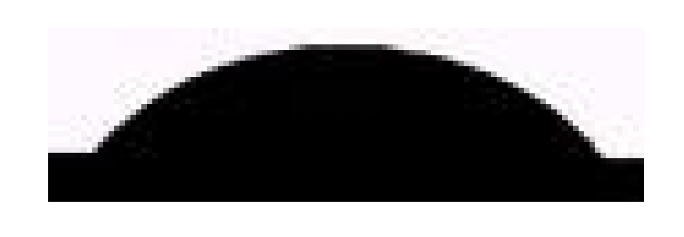	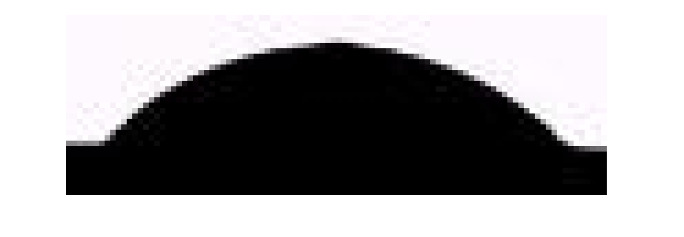	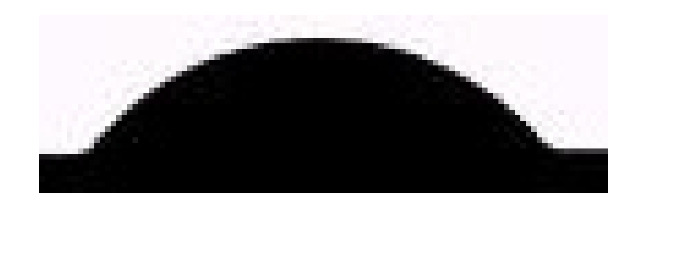	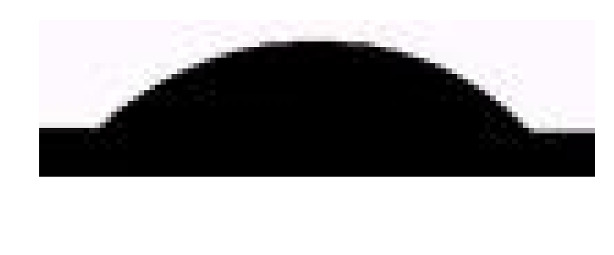	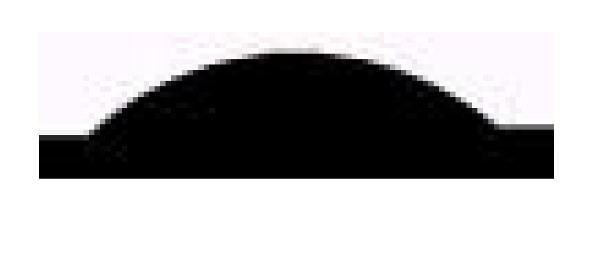
Surface free energy/mJ·m^−2^	47.48	38.99	33.64	37.45	47.85	49.32
Polar components /mJ·m^−2^	38.45	25.57	13.88	3.68	1.05	0.81
Dispersion component /mJ·m^−2^	9.03	13.43	19.76	33.77	46.80	48.52

**Table 3:** Element contents and O/C values of the control sample and vacuum heat-treated samples of bamboo.

Samples	C1s (%)	O1s (%)	O/C (%)
Untreated	68.37	31.63	46.26
140 °C	68.86	31.14	45.22
160 °C	70.51	29.49	41.82
180 °C	69.23	30.77	44.45
200 °C	70.83	29.17	41.18
220 °C	71.93	28.07	39.02

**Table 4:** Percentage of carbon in different chemical states of bamboo fiber before and after vacuum heat treatment.

Sample Type	The Peak Position (eV)				Relative Content (%)			
	C1	C2	C3	C4	C1	C2	C3	C4
Untreated	284.16	285.81	287.15	288.26	47.72	38.83	8.33	5.13
140 °C	284.19	285.80	287.10	289.25	47.37	34.25	18.30	0.07
160 °C	284.10	285.78	287.62	289.25	46.13	43.03	10.15	0.69
180 °C	284.12	285.82	287.38	289.25	45.11	40.45	14.34	0.11
200 °C	284.14	285.78	287.44	289.25	49.62	36.79	13.58	0.01
220 °C	284.13	285.82	287.70	289.25	51.58	37.54	10.55	0.33

**Table 5:** Contact angle and surface free energy of BF/PE composites.

Sample Type	Water Contact Angle/°		Glycol Contact Angle/°		Dispersion Component/mJ·m^−2^	Polar Component/mJ·m^−2^	Surface Free Energy /mJ·m^−2^
BF/PE /MAPE	86.00	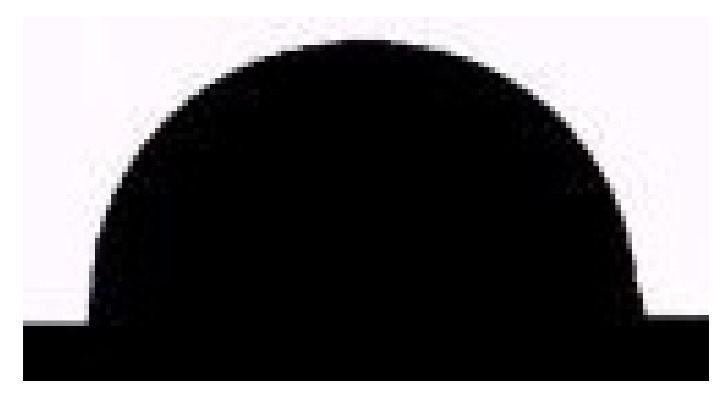	70.00	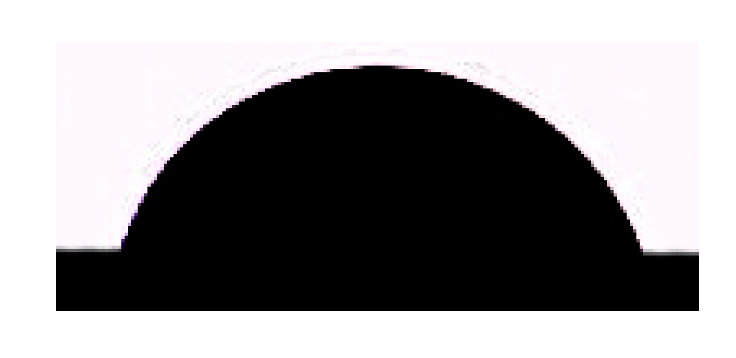	10.77	45.84	56.61
140 °C HTBF/PE /MAPE	87.00	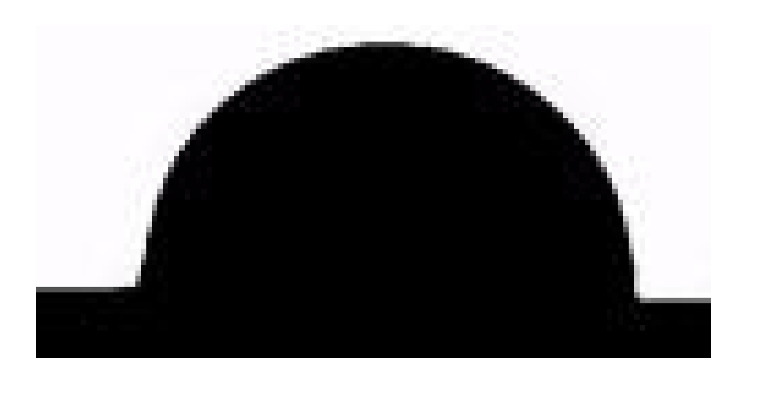	71.50	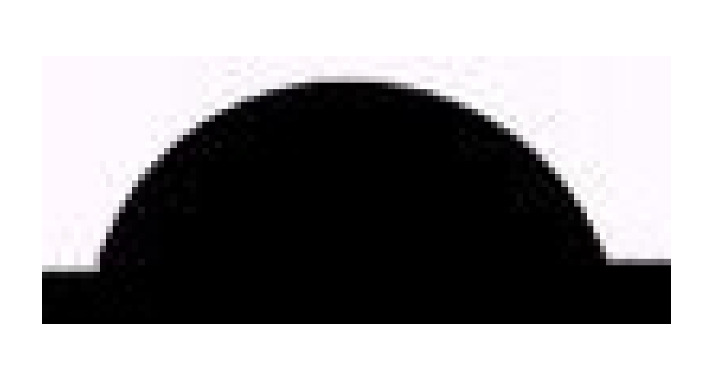	10.26	43.65	53.91
160 °C HTBF/PE/MAPE	87.50	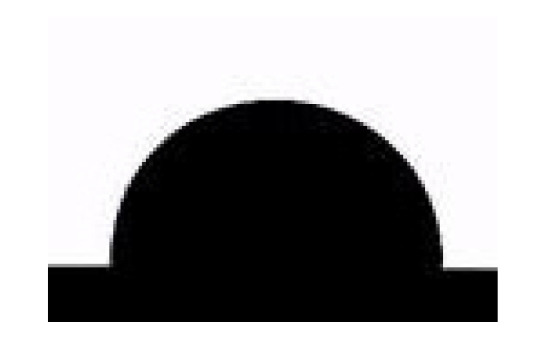	72.50	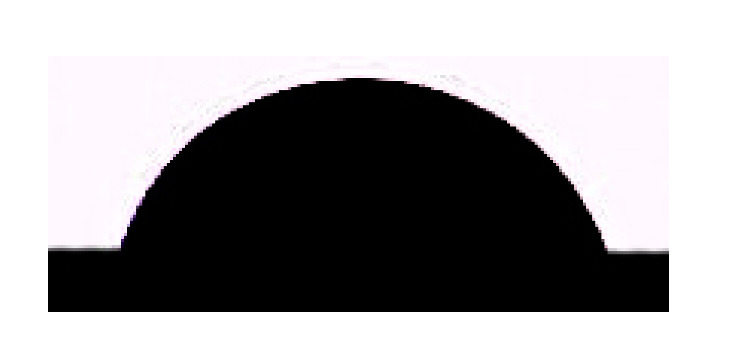	9.76	41.55	51.31
180 °C HTBF/PE /MAPE	88.00	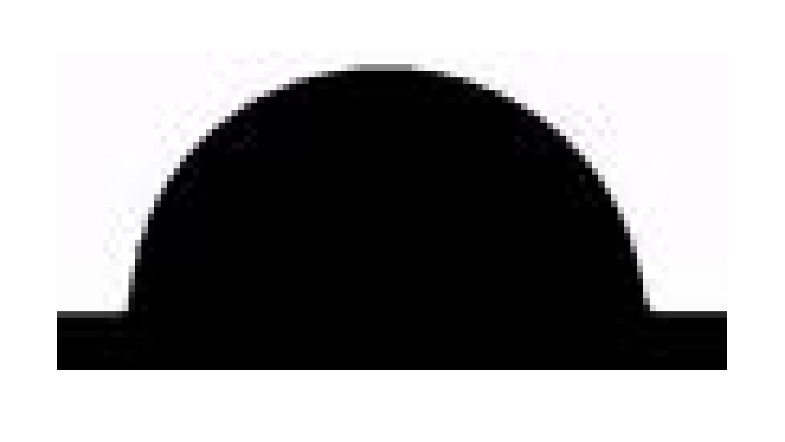	75.00	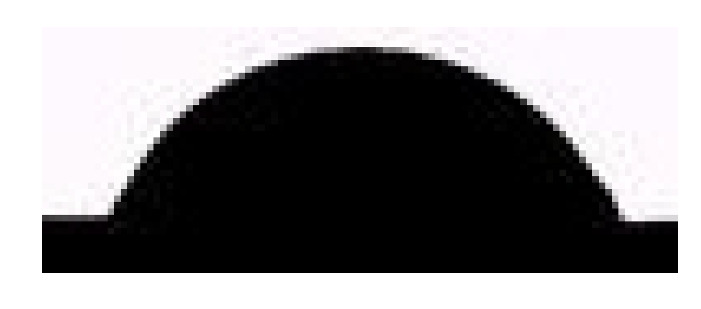	7.94	33.79	41.73
200 °C HTBF/PE /MAPE	85.00	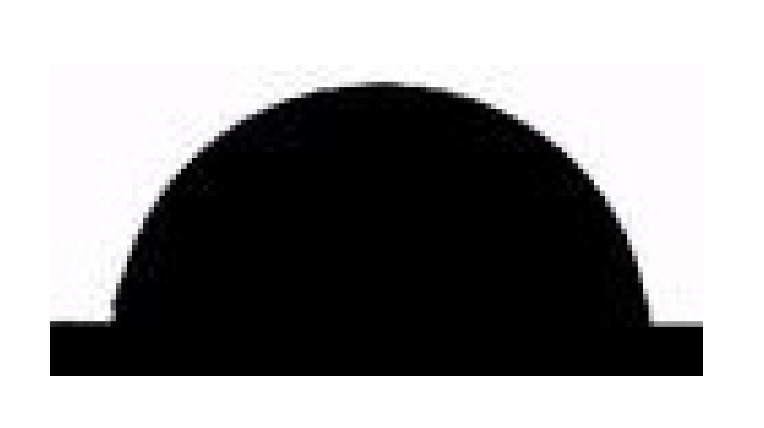	70.50	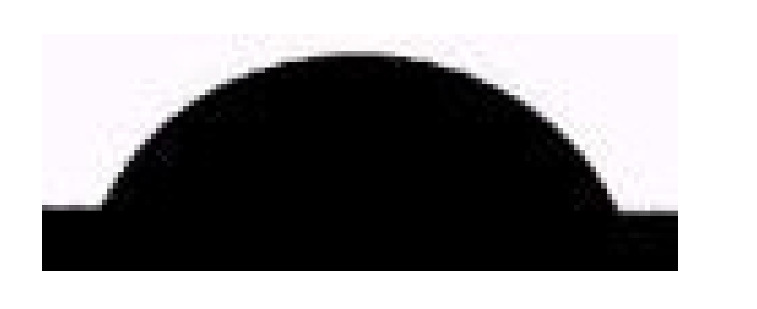	9.38	39.94	49.32
220 °C HTBF/PE /MAPE	83.50	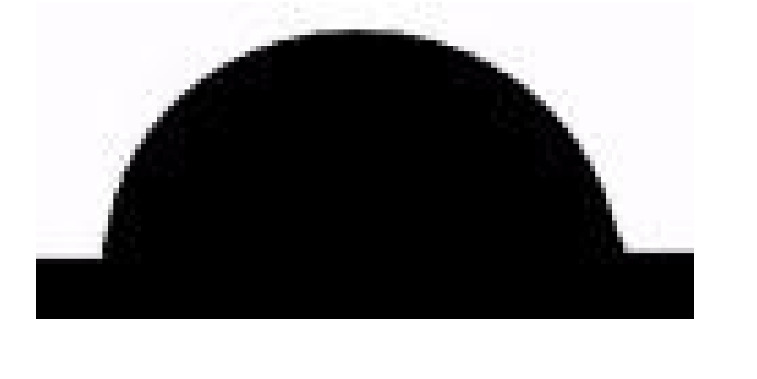	69.50	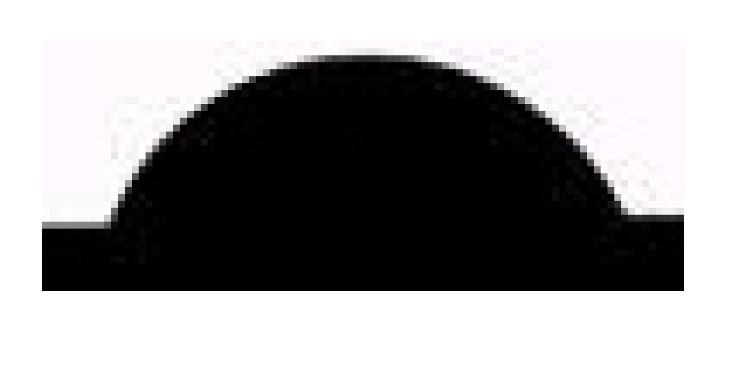	8.98	38.20	47.18

**Table 6:** Decomposition characteristic parameters of PE and bamboo fiber/PE composites.

Sample Type	Stage 2			Stage 3			Char Residue at 700 °C/wt%
	Onset Point/°C	T_max_/°C	V_max_/(% min^−^^1^)	Onset Point/°C	T_max_/°C	V_max_/(% min^−^^1^)	
PE	-	-	-	451.7	472.4	20.90	1.57
BF/PE	293.9	351.6	3.26	464.3	484.8	18.20	7.66
BF/PE/MAPE	294.9	352.2	3.18	467.0	487.2	18.75	7.19
140 °C HTBF/PE/MAPE	293.2	350.5	2.90	466.1	486.5	19.56	4.70
160 °C HTBF/PE/MAPE	294.3	352.9	3.31	466.4	486.5	18.21	7.56
180 °C HTBF/PE/MAPE	294.1	353.1	3.21	467.3	487.6	18.85	7.39
200 °C HTBF/PE/MAPE	295.3	352.2	3.30	468.3	488.2	19.13	7.54
220 °C HTBF/PE/MAPE	301.8	352.7	3.97	466.6	486.5	17.95	7.45

## Data Availability

All the data is provided in the manuscript.
